# A Hardware Pseudo-Random Number Generator Using Stochastic Computing and Logistic Map

**DOI:** 10.3390/mi12010031

**Published:** 2020-12-30

**Authors:** Junxiu Liu, Zhewei Liang, Yuling Luo, Lvchen Cao, Shunsheng Zhang, Yanhu Wang, Su Yang

**Affiliations:** 1 School of Electronic Engineering, Guangxi Normal University, Guilin 541004, China; j.liu@ieee.org (J.L.); lzw.soldier@gmail.com (Z.L.); 18810775110@163.com (L.C.); shunszhang@163.com (S.Z.); yanhu_huny1010@163.com (Y.W.); 2 Guangxi Key Lab of Multi-Source Information Mining & Security, Guangxi Normal University, Guilin 541004, China; 3 School of Computing and Engineering, University of West London, London W5 5RF, UK; scott.yang@uwl.ac.uk

**Keywords:** stochastic computing, chaos, logistic map, FPGA

## Abstract

Recent research showed that the chaotic maps are considered as alternative methods for generating pseudo-random numbers, and various approaches have been proposed for the corresponding hardware implementations. In this work, an efficient hardware pseudo-random number generator (PRNG) is proposed, where the one-dimensional logistic map is optimised by using the perturbation operation which effectively reduces the degradation of digital chaos. By employing stochastic computing, a hardware PRNG is designed with relatively low hardware utilisation. The proposed hardware PRNG is implemented by using a Field Programmable Gate Array device. Results show that the chaotic map achieves good security performance by using the perturbation operations and the generated pseudo-random numbers pass the TestU01 test and the NIST SP 800-22 test. Most importantly, it also saves 89% of hardware resources compared to conventional approaches.

## 1. Introduction

Pseudo-random numbers have an overwhelming impact on many fields, such as cryptography, digital signatures, image authentication watermarks, and protected communication protocols. The design of pseudo-random number generators (PRNGs) has greatly attracted the attention of many researchers. For the generation of pseudo-random numbers, PRNGs are usually based on the implementation of mathematical algorithms. Popular pseudo-random number generation methods include the mid-square method, linear congruence method, linear and nonlinear feedback shift registers [1]. However, due to the fixed linear structure inside, they are easy to be tracked and predicted, which usually leads to the system being not safe enough. The chaos-based system is highly sensitive to initial conditions and parameters. It has the characteristics of pseudo-randomness and unpredictability, which play an important role in information encryption. Therefore, chaotic systems are considered as an effective way to improve the performance of PRNGs.

Using chaotic systems to design a PRNG was first proposed in [2]. Subsequently, a lot of PRNGs based on chaotic systems were proposed. A method of designing PRNG based on the logistic map is proposed in [3]. Compared to the pseudo-random numbers produced by the congruence method, the random numbers generated by the logistic map is large and aperiodic which appears to have better performance. The PRNG construction model based on the discrete chaotic dynamic system is proposed in [4]. In [5], a PRNG based on the piecewise chaos-based system is proposed. In [6], a unidirectional coupling map lattice composed of logical maps is used to construct a spatiotemporal chaotic system. On this basis, a PRNG based on the spatiotemporal chaos-based map is proposed. In [7], two PRNGs were constructed using 2D dynamic systems constituted by two symmetrically coupled logical maps. The design of a double chaotic system based on logistic map is proposed in [8], and PRNG is derived from random independent initial conditions. In [9], a PRNG based on the composition of several tent maps with diverse initial parameters is proposed, and it can be further used to develop chaotic stream cyphers. A nonlinear principle is proposed in [10] to generate pseudo-random bit sequences. During the generation process, the parameter of a chaotic map is used to disturb the trajectory of another chaotic map to extend its period length. In [11], a PRNG was proposed based on the three-dimensional Chen chaotic system. In [12], an algorithm for generating multiple pseudo-random sequences using chaotic functions was developed. The initial values of the chaotic system are calculated and indexed by a based-chaos linear congruences function.

All of the above methods are chaos-based PRNGs, and are all realized using software. In addition, many researchers have contributions in hardware implementation of chaos-based PRNGs. In [13], fully digital circuits are used to implement a chaos-based PRNG, and the clock frequency achieves 120 Mhz. In [14], the spatiotemporal chaotic system is digitized, and the highly parallel PRNGs are implemented on the Field Programmable Gate Arrays (FPGAs). In [15], one-dimensional, two-dimensional, and three-dimensional chaotic systems are implemented and verified on the FPGA platform. In [16], a post-processing technique for chaotic mapping in digital systems is proposed, which supports the third-order chaotic system. The chaotic degradation of digital chaotic systems is greatly reduced. In [17], the optimal parameters of the four chaotic maps are analyzed, and the PRNGs are implemented on the FPGA platform using floating point number and fixed-point number, respectively. In [18], the period of digital chaotic mapping is extended by a simple recursive structure and perturbation, and this PRNG is with low-complexity and long-period safety.

Compared with Application-specific Integrated Circuits, FPGAs have the characteristics of high program flexibility and high parallel computing efficiency. Thus, FPGA is usually selected as the platform for digital implementation of PRNG. However, in practical applications, the hardware resources are limited, and PRNG based on chaotic mapping consume more resources, especially the Digital Signal Processor (DSP) resources. Stochastic computing (SC) is based on a form of probability, which optimizes the hardware implementation of PRNG in this paper. Traditionally, the numbers of SC are substituted by the statistical distribution of the stochastic bit stream. The value of traditional number is converted into stochastic bit stream to represent the probability value in the interval [0, 1]. Then a simple gate circuit can be used to perform arithmetic operations of classic calculations. This characteristic allows SC to provide higher fault tolerance and lower hardware resource dissipation, and it can keep almost the same computing performance like conventional computing technology [19]. In this paper, the arithmetic operations of chaotic systems are replaced by the compact hardware of SC to reduce the computational complexity. For the computing characteristics of the digital platform and SC, the logistic map has been changed accordingly as well as its arithmetic expression. The hardware implementation of the computing unit of PRNG is essentially simplified, which maintains low power and resource dissipation. The main contribution of this paper is that the SC method is used to optimize the digital implementation of PRNG, and the performance analysis and hardware resource consumption are given.

The rest of this paper is organized as follows. The background is retrospect in Section 2. The basic mechanism of the proposed chaotic PRBG is presented in Section 3. Section 4 analyses the chaotic properties of the improved logistic map. Section 5 concludes this paper.

## 2. Background

We review the related works of the logistic map and stochastic computing in this section.

### 2.1. Logistic Map

**Figure 1:**
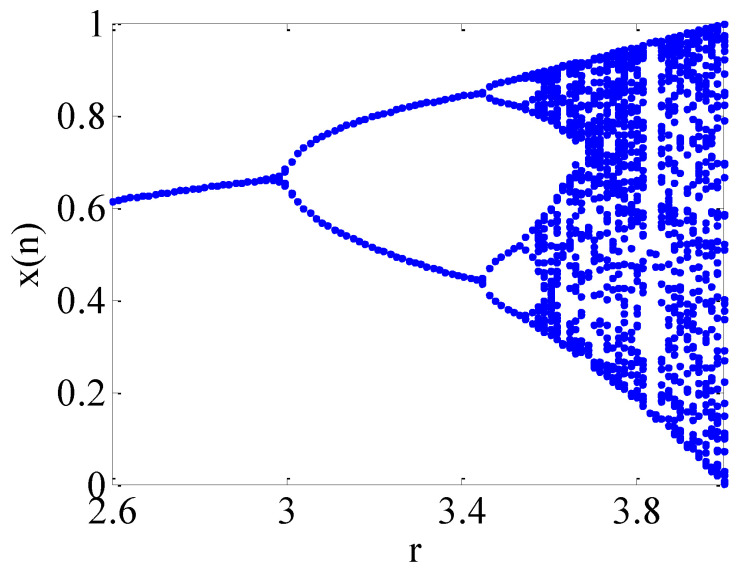
Logistic map.

The one-dimensional logistic map [20] is a nonlinear discrete system, which is easy to be implemented in hardware due to its simplicity. It is one of the most widely used chaotic maps. Its iterative equation is defined by

(1) $$\begin{array}{c} x_{n+1}=rx_{n}\left(1-x_{n}\right), \end{array}$$

where the range of control parameter *r* is 0<r≤4, and the state value for each iteration is $0<x_{n}<4$. The initial value of the iteration is $n=1,2,3,\ldots ,x_{n}$. When the control parameter *r* changes, the behaviour of the logistic map will be very different. Figure 1 depicts the bifurcation diagram of the logistic map when the initial value is 0.45. It describes the distribution of the iteration values as a function of the parameter *r*. The abscissa is the parameter *r*, and the ordinate is the iterative state value $x_{n}$. When parameter *r* increases, the mapping iteration motion will appear in multiple states. When 3.569945672<r≤4, the values of $x_{n}$ will be distributed within a certain range, and there is no fixed period. In the meanwhile, the logistic map exhibits a chaotic state.

**Figure 2:**
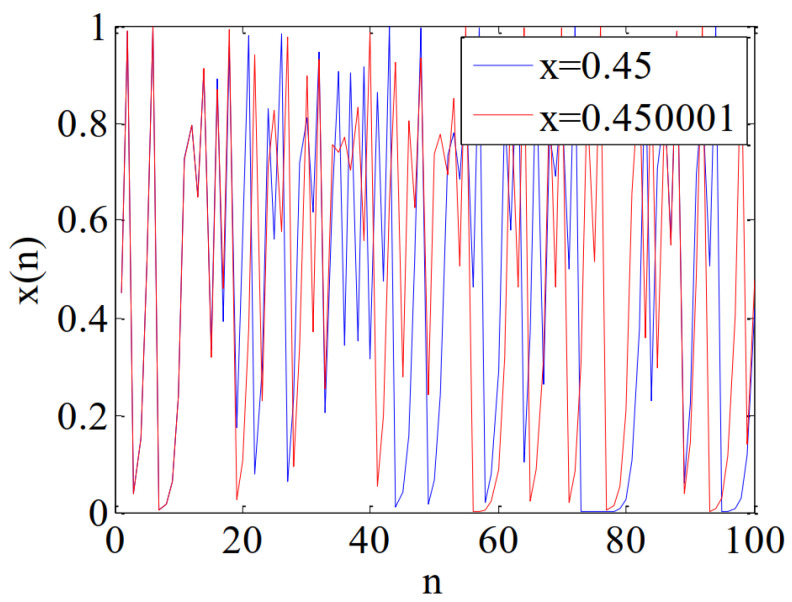
Chaotic sequences.

The chaotic map is highly sensitive to the initial value [21]. The logistic map is iterated 100 times when the control parameter *r* is 4, and the initial values are $x_{0}=0.45$ and $x_{0}=0.450001$. The results are shown in Figure 2, and it demonstrates the initial condition sensitivity of logistic map. The two chaotic systems are only with extremely small differences in initial values. In the iterative process, the errors are rapidly amplified and the trajectories become irrelevant, which make it extremely difficult to crack the logistic map based encryption system.

### 2.2. Stochastic Computing

The basic rule of SC is that the calculated data are presented in a stochastic bit stream, and then the data are processed in the form of digital probability. There are two representations for converting traditional numbers to a stochastic bit stream: unipolar and bipolar [22]. The number range of unipolar representation is [0, 1] and bipolar is [−1, 1]. In SC, the number represented by the stochastic bit stream is replaced by the probability of occurrence of “1” in this bit stream. For example, three different stochastic bit streams (1,0,0,0), (0,1,0,0) and (0,1,0,0,0,1,0,0) in unipolar. In the representation, the number 0.25 is indicated. For example, there are three different stochastic bit streams (0,0,1,0), (1,0,0,0) and (1,0,0,0,0,0,0,1), and they all represent the number 0.25 in unipolar notation. These bit streams represent the number −0.5 in bipolar. The number represented by the unipolar stochastic bit stream can be expressed by the following formula:

(2) $$\begin{array}{c} X=P(X=1)=P(X), \end{array}$$

where *X* is the value of a traditional number, and P(X=1) is the probability of the occurrence of “1” in the random bit stream *X* and is represented by P(X). The number formula represented by the bipolar stochastic bit stream is:

(3) $$\begin{array}{c} X=2*P(X=1)-1=2*P(X)-1. \end{array}$$

**Figure 3:**
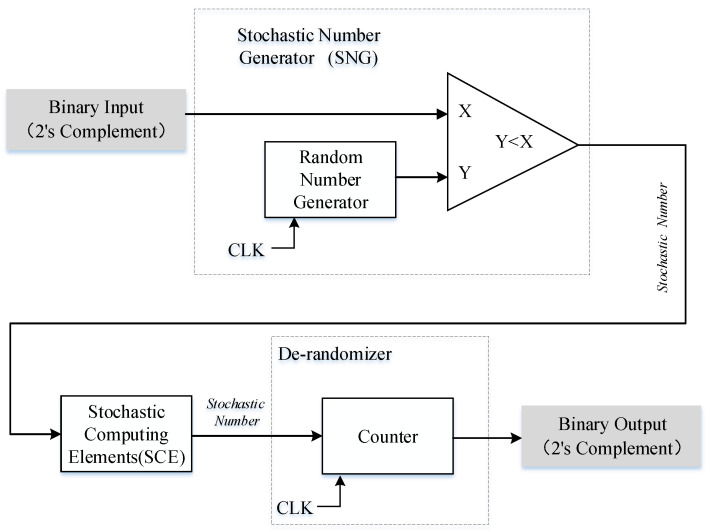
The structure of stochastic computing.

**Figure 4:**
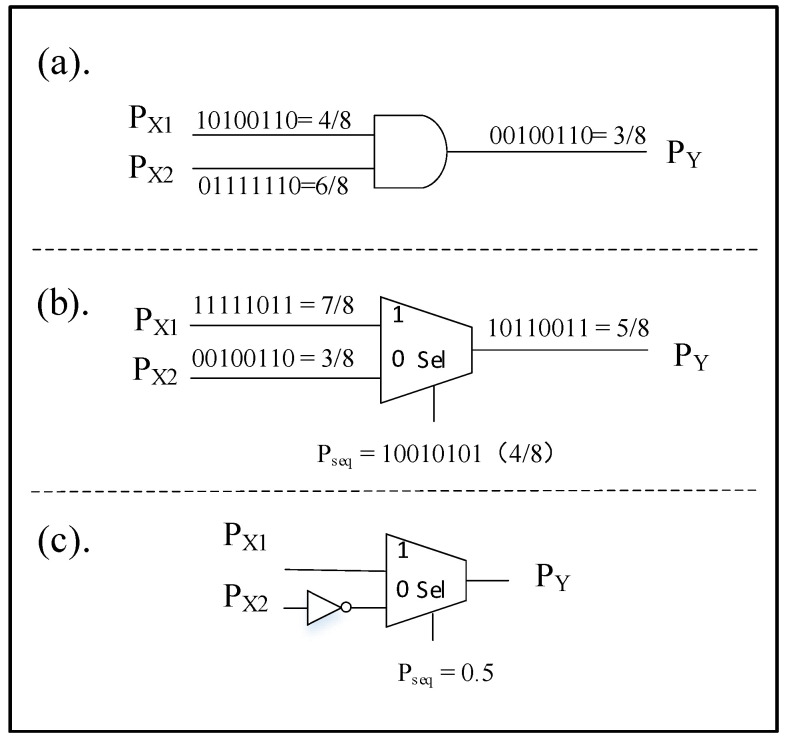
The circuit structure of SCE. (**a**) is a multiplier, (**b**) is an adder, and (**c**) is a subtractor.

The architecture of SC is shown in Figure 3, which usually consists of three main components: stochastic number generator (SNG), stochastic computing element (SCE), and a de-randomizer [23]. The SNG is implemented by using a random number generator and a comparator. On the FPGA platform, we can use a linear feedback shift register (LFSR) instead of the random number generator. SNG is used to convert the binary value into random bit stream. At the same time, a de-randomizer of a binary counter is usually used to decode the output stochastic bit stream into a deterministic binary number [19]. The SCE is an arithmetic operator part of SC, such as multiplier, adder or subtractor. In this section, only unipolar SCE is introduced, because this article only uses a unipolar operation. The circuit structure of SCE is shown in Figure 4.

(a). ***Multiplication operation***. In SC, the multiplication of two stochastic bit streams can be realized by a simple gate circuit, which can save a lot of hardware resources in large-scale calculations. Unipolar multiplication only needs to pass the input bit streams through an AND gate to get the output. It should be noted that the two input bit streams must be guaranteed to be uncorrelated.

(b). ***Addition operation***. In SC, the unipolar bit stream represents values between [0, 1]. The numerical range of the result after the addition should be [0, 2], which is not within the indicated range, so the addition in the random calculation needs to be performed by a special operation, which is called as scaled addition. The scaled addition operation allows the multiplexer to scale the output to the normal range.

(c). ***Subtraction operation***. Its circuit structure is almost the same as an addition operation, where only a NOT gate is used between the second bitstream and the selector.

## 3. Enhanced Digital Logistic Map and Hardware Implementation

In this section, an enhanced logistic map is introduced, and it is used to reduce the chaotic degradation caused by digital implementation. Then the mathematical expression of the logistic map is transformed to fit the characteristics of the SC. Finally, the hardware implementation of PRNG is given.

### 3.1. Enhanced Digital Logistic Map

When the logistic map is in real continuous field, a non-periodic unpredictable sequence can be generated after giving the initial value $x_{0}$ and the control parameter *r*, and r∈(3.569945672,4]. The classical SC has a calculation range of [0, 1]. For a logistic map, only the control parameter *r* is not within this range, which cannot be represented by SC. Thus, we change the mathematical expression of logistic, and it is shown as

(4) $$\begin{array}{cc} x_{n+1} & =rx_{n}\left(1-x_{n}\right)\\ \; & =\left(4-d\right)x_{n}\left(1-x_{n}\right)\\ \; & =4x_{n}\left(1-x_{n}\right)-dx_{n}\left(1-x_{n}\right), \end{array}$$

where r=4−d, then d=4−r∈[0,0.430054328). In this manner, a new control parameter can be denoted as *d*, and all data can be converted into the form of SC.

However, due to the limited precision of a digital system, the chaotic system will generate quantization error in the digital realization process, and the pseudo randomness of the chaotic system is degraded [24]. If the calculation precision is a *L*-bit binary number, the data space is $2^{L}$. The number of generated iteration values can only be $2^{L}$, which is a finite precision due to the digitization. The problems of short period and high autocorrelation will reduce the performance of PRNG. Thus, if only using chaotic systems, the performance of PRNG cannot meet the requirements of practical applications.

**Figure 5:**
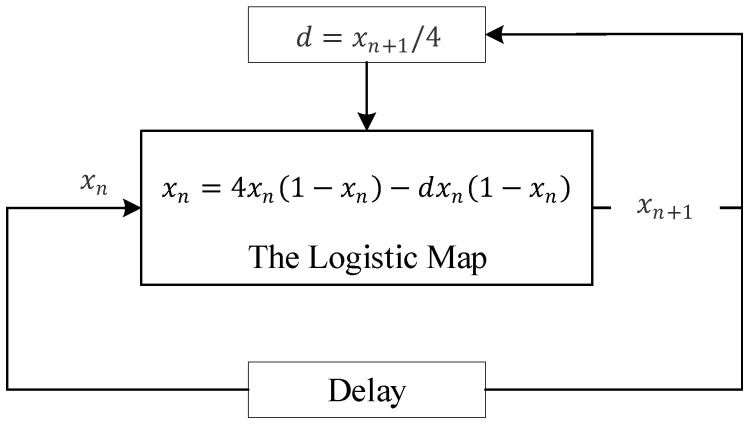
The block diagram of the enhanced logistic map.

There are five main ways to promote the degradation of chaotic maps on digital systems: (a) using higher precision [25]. This kind of scheme can prevent the degradation to a certain extent, but the computation costs will grow geometrically, which will affect the speed and consume much more resources when the chaotic system is implemented by hardware. (b) Cascading multiple chaotic systems [26]. This scheme can efficiently extend the period of the digital chaos-based system but may result in poor data distribution. (c) Multiple chaotic system switching [27]. It is often difficult to design an effective switching strategy between multiple chaotic systems. (d) Error compensation method [28]. This scheme can effectively improve the performance of digital chaotic systems, but it is not easy to be extended to high-dimensional chaotic systems. (e) Disturbance methodology [29]. This methodology can prevent the degradation of digital chaotic systems. Disturbance objects including input, output, and system parameters. If the perturbation algorithm is well designed, good performance can be obtained. In this paper, the perturbation methodology is adopted, and we select to disturb the parameter *d*. The block diagram of the system is shown in Figure 5. The initial value of the parameter *d* is set to 0. The *d* in the subsequent iteration is determined by the output of the previous iteration. The output is disturbed to control the parameter *d* by dividing, and *d* is controlled within the corresponding range.

### 3.2. Hardware Implementation of PRNG

**Figure 6:**
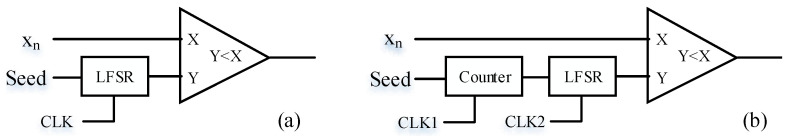
The circuit structure of SNG. (**a**) The conventional SNG; (**b**) The improved SNG.

Considering the complexity of hardware implementation, SC is used in this paper to replace the arithmetic unit of traditional digital circuits. In the SC, it is first necessary to convert the fixed-point fraction to the stochastic number through SNG. The structure of the SNG is shown in Figure 6. Figure 6a shows the structure of a conventional SNG. $x_{n}$ is a fixed-point number, and it is compared with a LFSR which has the same number of bits. If the value of the LFSR is greater than $x_{n}$, the value of the stochastic sequence at the current time is 1, otherwise, it is 0. Finally, a complete stochastic number is generated. Figure 6b shows the improved SNG structure. When the rising edge of CLK2 is detected, LFSR generates a stochastic number. The seed of the LFSR is set to be variable. After the LFSR runs for one cycle, the CLK1 becomes high, and value of the seed is increased, seed=seed+1. Even if the chaotic system generates the same iteration value, the stochastic number of SNG conversion will not be the same. The above operation acts as a minor disturbance to prevent chaotic systems from falling into a short cycle.

**Figure 7:**
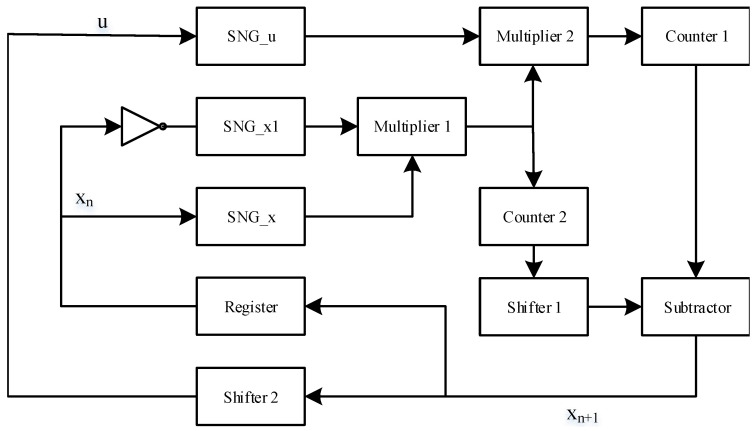
The hardware structure of the PRNG based on the enhanced logistic map.

The hardware structure of the enhanced logistic map is shown in Figure 7. The implementation accuracy is determined by the period length of the LFSR. In Figure 7, a NOT gate is added between SNG_x1 and input $x_{n}$, and the resulting stochastic number represents $1-x_{n}$. SNG_x produces a stochastic number $x_{n}$. Counter represents a decoder in SC that converts a stochastic number into a fixed-point fractional form. The function of Shifter1 is to shift the value of $\left(1-x_{n}\right)*x_{n}$ to the left by two bits. Then, the final output value $x_{n+1}$ is obtained by subtracting the output value $u*x_{n}*\times \left(1-x_{n}\right)$ of Shifter1 from the output value $4*x_{n}*\times \left(1-x_{n}\right)$ of counter1. The function of Shifter2 is to right shift $x_{n+1}$ by two bits. The output value is in the range of (0, 0.25), and it will be used as the control parameter *d* for the next iteration.

## 4. Performance Analysis

### 4.1. Performance Improvement of Digital Chaotic System

**Figure 8:**
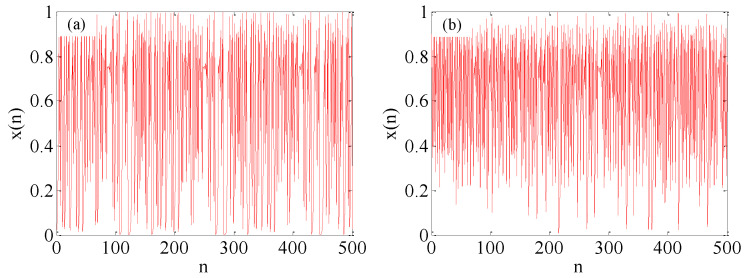
Iterative values produced by the logistic map and the proposed method based on digital implementation. (**a**) The original logistic map; (**b**) The enhanced logistic map.

If the precision is limited when implementing a chaotic system, the short cycle phenomenon will occur. The data precision and pseudo-random number generation rate in SC depends on the length of the stochastic sequence, while the length of the stochastic sequence depends on the period length of the LFSR [22]. Specifically, the longer the sequence length of the LFSR, the higher the numerical accuracy and more hardware resources it will consume. Therefore, to achieve a trade-off between the numeric accuracy and hardware resource consumption, the 16-bit LFSR is used to generate random numbers, and the working frequency of proposed system is 100 MHz, which represents the pseudo-random number generation rate is 1.5 KHz, and the effective precision of the fixed-point number is 16 bits. As shown in Figure 8a, the motion trace of the original logical map enters a loop after several iterations. On the contrary, the enhanced logistic map avoids the short period, and the improved system exhibits better randomness, as shown in Figure 8b.

### 4.2. Initial Value Sensitivity

**Figure 9:**
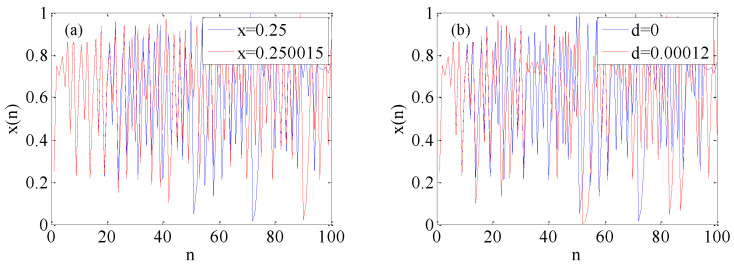
Sequences generated by enhanced logistic map using different initial values. (**a**) Initial values of 0.25 and 0.250015; (**b**) Initial values of 0 and 0.00012.

Initial value sensitivity is an indispensable indicator of PRNGs. After several iterations, the motion trajectories of the chaotic system will be very different if there are extremely small changes in initial values. The control parameter d=0 in the enhanced logistic map is kept unchanged, and the initial values of the system are taken as $x_{0}=0.25$ and $x_{0}=0.250015$, respectively. The changes of the two sequences after 100 times of iterations are as shown in Figure 9a. The trajectories of the chaotic system have a huge difference after several iterations. Similarly, when the initial value x=0.25 is kept unchanged, a slight variation is given to the initial parameters. The generated sequences are shown in Figure 9b. When the system goes through several iterations, the trajectories show a huge difference. The result indicates that the enhanced logistic map has good initial sensitivity.

### 4.3. Chaotic Attractor

**Figure 10:**
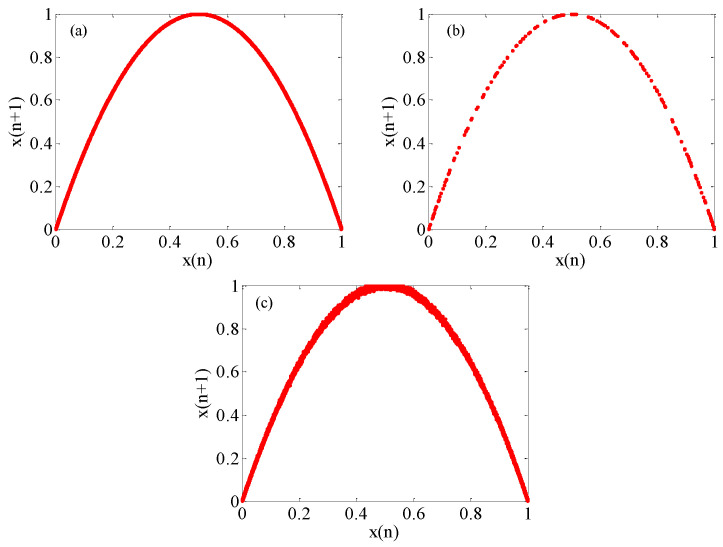
Comparison of chaotic attractors. (**a**) The original logistic map; (**b**) The logistic map of getting stuck in cycles; (**c**) The enhanced logistic map.

Chaotic attractors usually have a fixed geometric structure, and the structural complexity of the attractor reflects the chaotic degree. In this experiment, 10,000 continuous values were selected for generating the chaotic attractors. Figure 10a shows the chaotic attractor of the original logistic map. Its orbit is in the interval of (0, 1). For the digital realization with limited precision, the logistic map gets stuck in cycles and the iterative sequence cannot achieve full traversal, as shown in Figure 10b. However, after the perturbation, the enhanced logistic map is traversed, as shown in Figure 10c. This chaotic attractor is not a strict curve, but a thicker curve oscillates around it. This is better than the original situation, since the orbit is traversal and more complex.

### 4.4. Autocorrelation

**Figure 11:**
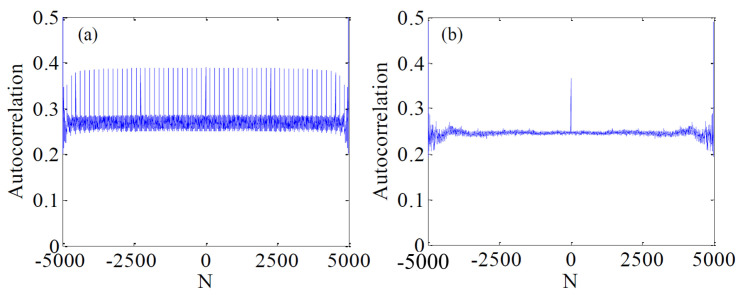
Autocorrelation comparison based on different logistic maps. (**a**) The original logistic map; (**b**) The enhanced logistic map.

Autocorrelation is used to describe the correlation between values at different times in a sequence [30]. Ideally, the autocorrelation of any random sequence is an impulse function. Figure 11a shows the autocorrelation of the output sequence by original logistic map. The correlation between adjacent tracks is strong, which makes the system vulnerable to attack. Figure 11b is the improved autocorrelation, and it shows that the autocorrelation in a sequence is greatly reduced.

### 4.5. Approximate Entropy

Approximate entropy is often utilized to measure the randomness of binary sequences. According to [31], the approximate entropies of the sequences produced by three systems are obtained and shown in Table 1. The iteration values between the tent map and logistic map are generated on the platform of Matlab 2014a. The parameter of tent map is set as 0.3, and the logistic map parameter is set as 4. The iterative values of the proposed system is calculated by the SC. The approximate entropy based on the proposed system is improved compared with the original logistic map.

### 4.6. Histogram of Frequency Distribution

**Figure 12:**
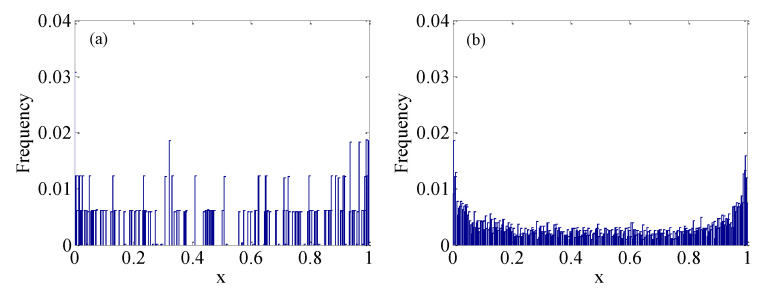
Histogram comparison based on different logistic maps. (**a**) The original logistic map; (**b**) The enhanced logistic map.

The frequency histogram can reflect the distribution of the chaotic sequence. The frequency distribution of the original logic map is shown in Figure 12a, and the frequency distribution of the enhanced logistic map using the SC is shown in Figure 12b. The frequency distribution of Figure 12a is not ideal, since the distribution of values is not uniform, but an obvious improvement is obtained in Figure 12b. Thus, the system proposed in this paper can greatly enhance the ability to resist frequency attacks.

### 4.7. NIST SP 800-22 Analyses

The National Institute of Standards and Technology SP800-22 is a standard suite for measuring the randomness of binary sequences [32]. It consisted of 15 subtests, and these subtests are used to test the same sequence from different aspects. In this test, the data produced by the proposed system are 1,000,000 bits, and the significant level α is set as 0.01. When the P-value is greater than the significant level α, the test is passed, otherwise, the test fails. The analysis results are displayed in Table 2. It can be described that the stochastic bit stream produced by the proposed system has excellent statistical properties.

### 4.8. TestU01 Test

TestU01 [33] is also a commonly used random number test standard. It provides several sets of tests, in which each test contains different subtests. In this experiment, we mainly use the Rabbit and Alphabit tests. The data length is $2^{20}$ bits. Rabbit test has a total of 38 small tests under this data length, and Alphabit test contains 17 tests. These two tests are mainly used to test hardware implemented PRNGs. Table 3 shows the results of the TestU01 test, and the improved system passes all the tests. Thus, the proposed system has better randomness compared to the original logistic map.

### 4.9. Area Overhead

The proposed system is performed on the ZedBoard Zynq Evaluation and Development Kit (xc7z020clg484-1) platform, and its working frequency is 100 MHz, and the utilizations of all hardware resource consumption are summarized. The comparison of resource consumption is shown in Table 4, where 373 LUTs and 445 registers are consumed, and the utilization rates are 0.7% and 0.4% of the ZedBoard device, respectively. The PRNG in [34] also uses the logistic map. Compared to it, the consumption of look-up tables (LUTs) is reduced by 89.6%, the consumption of the register is reduced by 62.2%, and the DSP blocks are not used. Results show that the PRNG implemented on the FPGA combined with the SC and the enhanced logistic map occupies the least resources.

## 5. Conclusions

In this paper, a hardware PRNG based on the enhanced logistic map has been presented, and it is optimized by using the technique of SC. Compared with existing approaches, this proposed work reduces 89% of the consumption of hardware resources, especially its implementation does not require DSP blocks which are expensive for FPGA devices. Besides, the pseudo-random numbers generated by the proposed PRNG pass the TestU01 test and the NIST SP 800-22 test. The performance and statistical analysis results demonstrate a high-security performance of the proposed work with relatively low hardware utilizations. Future work will aim to further reduce the required hardware resource for complex digital chaotic system implementations.

## Figures and Tables

**Table 1:** Approximate entropy comparison based on three chaotic maps.

Chaotic System	Tent Map	Logistic Map	The Proposed System
Approximate entropy	0.5170	0.6506	0.7112

**Table 2:** Results of the NIST SP800-22 analyses.

Sub-Tests	*p*-Value	Result
Frequency	0.213309	Pass
Universal	0.554405	Pass
Serial	0.739918	Pass
Rank	0.441376	Pass
FFT	0.902014	Pass
Runs	0.350485	Pass
Longest Run	0.911413	Pass
Block Frequency	0.565945	Pass
Cumulative Sums	0.534146	Pass
Overlapping Template	0.395130	Pass
Non-overlapping Template	0.066882	Pass
Approximate Entropy	0.122325	Pass
Linear Complexity	0.911413	Pass
Random Excursions	0.652391	Pass
Random Excursions Variant	0.381832	Pass

**Table 3:** Results of the TestU01 test for different PRNGs.

PRNGs	Rabbit	Alphabit
Original logistic map	35/38	15/17
Enhanced logistic map	38/38	17/17

**Table 4:** Comparison of resource consumption based on different PRNGs.

Approaches	Chaotic Generators	LUTs	Registers	DSP Blocks	Frequency (MHz)	Device
[35]	Jerk	716	130	16	50.46	Virtex 4
[36]	Cubic map	387	N/A	24	100	Virtex 6
[34]	Logistic map	3711	1176	N/A	50	Cyclone II
[37]	Loren	3476	N/A	N/A	100	Virtex 6
[38]	Coupled map lattices	2548	2429	32	100	Stratix IV
This work	Logistic map	373	445	0	100	ZedBoard

## References

[1] Liu L., Miao S., Hu H., Deng Y. (2016). Pseudorandom bit generator based on non-stationary logistic maps. IET Inf. Secur..

[2] Oishi S., Inoue H. (1982). Pseudo-random number generators and chaos. Trans. Inst. Electron. Commun. Eng. Jpn..

[3] Andrecut M. (1998). Logistic map as a random number generator. Int. J. Mod. Phys. B.

[4] Shujun L., Xuanqin M., Yuanlong C. (2001). Pseudo-random bit generator based on couple chaotic systems and its applications in stream-cipher cryptography. Progress in Cryptology—INDOCRYPT 2001, Proceedings of the International Conference on Cryptology in India, Calcutta, India, 10–13 December 2001.

[5] Meng F.S., Yang C.Z., An Z.Y. (2004). Chaos-Based Random Number Generators. J. Comput. Res. Dev..

[6] Li P., Li Z., Halang W.A., Chen G. (2005). A novel multiple pseudo random bits generator based on spatiotemporal chaos. IFAC Proc. Vol..

[7] Pellicer-Lostao C., López-Ruiz R. Pseudo-random bit generation based on 2D chaotic maps of logistic type and its applications in chaotic cryptography. Proceedings of the International Conference on Computational Science and Its Applications.

[8] Patidar V., Sud K.K., Pareek N.K. (2009). A pseudo random bit generator based on chaotic logistic map and its statistical testing. Informatica.

[9] Cristina D.A., Radu B., Ciprian R. A new pseudorandom bit generator using compounded chaotic tent maps. Proceedings of the International Conference on Communications (COMM).

[10] Wang X.Y., Yang L. (2012). Design of pseudo-random bit generator based on chaotic maps. Int. J. Mod. Phys. B.

[11] Hu H., Liu L., Ding N. (2013). Pseudorandom sequence generator based on the Chen chaotic system. Comput. Phys. Commun..

[12] Francois M., Grosges T., Barchiesi D., Erra R. (2013). A new pseudo-random number generator based on two chaotic maps. Informatica.

[13] Yang H.T., Huang J.R., Chang T.Y. A chaos-based fully digital 120 MHz pseudo random number generator. Proceedings of the IEEE Asia-Pacific Conference on Circuits and Systems.

[14] Mao Y., Cao L., Liu W. Design and FPGA implementation of a pseudo-random bit sequence generator using spatiotemporal chaos. Proceedings of the International Conference on Communications, Circuits and Systems.

[15] Mansingka A.S., Radwan A.G., Salama K.N. Fully digital 1-D, 2-D and 3-D multiscroll chaos as hardware pseudo random number generators. Proceedings of the International Midwest Symposium on Circuits and Systems (MWSCAS).

[16] Barakat M.L., Mansingka A.S., Radwan A.G., Salama K.N. (2013). Generalized Hardware Post-processing Technique for Chaos-Based Pseudorandom Number Generators. ETRI J..

[17] de la Fraga L.G., Torres-Pérez E., Tlelo-Cuautle E., Mancillas-López C. (2017). Hardware implementation of pseudo-random number generators based on chaotic maps. Nonlinear Dyn..

[18] Dastgheib M.A., Farhang M. (2017). A digital pseudo-random number generator based on sawtooth chaotic map with a guaranteed enhanced period. Nonlinear Dyn..

[19] Brown B.D., Card H.C. (2001). Stochastic neural computation. I. Computational elements. IEEE Trans. Comput..

[20] Boeing G. (2016). Visual analysis of nonlinear dynamical systems: Chaos, fractals, self-similarity and the limits of prediction. Systems.

[21] Luo Y., Ouyang X., Liu J., Cao L. (2019). An image encryption method based on elliptic curve elgamal encryption and chaotic systems. IEEE Access.

[22] Alaghi A., Hayes J.P. (2013). Survey of stochastic computing. ACM Trans. Embed. Comput. Syst. TECS.

[23] Wong M.M., Wong D.M.L. (2017). Stochastic computing with spiking neural P systems. J. Univ. Comput. Sci..

[24] Ouyang X., Luo Y., Liu J., Liu Y., Bi J., Qiu S. Period analysis of chaotic systems under finite precisions. Proceedings of the International Conference on Systems Engineering (ICSEng).

[25] Wheeler D.D., Matthews R.A. (1991). Supercomputer investigations of a chaotic encryption algorithm. Cryptologia.

[26] Heidari-Bateni G., McGillem C.D. (1994). A chaotic direct-sequence spread-spectrum communication system. IEEE Trans. Commun..

[27] Wong K.W., Kwok B.S.H., Law W.S. (2008). A fast image encryption scheme based on chaotic standard map. Phys. Lett. A.

[28] Hu H., Xu Y., Zhu Z. (2008). A method of improving the properties of digital chaotic system. Chaos Solitons Fractals.

[29] Xing-Yuan W., Lin-Lin W. (2011). A new perturbation method to the Tent map and its application. Chin. Phys. B.

[30] Yoshioka D., Tsuneda A. Design of pseudochaotic maximum length sequences with prescribed autocorrelation obtained from discretized chaos maps. Proceedings of the International Symposium on Spread Spectrum Techniques and Applications.

[31] Pincus S.M. (1991). Approximate entropy as a measure of system complexity. Proc. Natl. Acad. Sci. USA.

[32] Bassham L.E., Rukhin A.L., Soto J., Nechvatal J.R., Smid M.E., Barker E.B., Leigh S.D., Levenson M., Vangel M., Banks D.L. (2010). Sp 800-22 rev. 1a. a Statistical Test Suite for Random and Pseudorandom Number Generators for Cryptographic Applications.

[33] L’Ecuyer P., Simard R. (2007). TestU01: AC library for empirical testing of random number generators. ACM Trans. Math. Softw. TOMS.

[34] Hue T.T.K., Van Lam C., Hoang T.M., Al Assad S. Implementation of secure SPN chaos-based cryptosystem on FPGA. Proceedings of the International Symposium on Signal Processing and Information Technology (ISSPIT).

[35] Mansingka A.S., Radwan A.G., Salama K.N. Design, implementation and analysis of fully digital 1-D controllable multiscroll chaos. Proceedings of the ICM 2011 Proceeding.

[36] Giard P., Kaddoum G., Gagnon F., Thibeault C. FPGA implementation and evaluation of discrete-time chaotic generators circuits. Proceedings of the Conference on IEEE Industrial Electronics Society.

[37] Chung H., Miri A. On the hardware design and implementation of a chaos-based RFID authentication and watermarking scheme. Proceedings of the International Conference on Information Science, Signal Processing and their Applications (ISSPA).

[38] Cao L., Luo Y., Bi J., Qiu S., Lu Z., Harkin J., McDaid L. (2015). An authentication strategy based on spatiotemporal chaos for software copyright protection. Secur. Commun. Netw..

